# Scalable quality control on processing of large diffusion-weighted and structural magnetic resonance imaging datasets

**DOI:** 10.1371/journal.pone.0327388

**Published:** 2025-08-01

**Authors:** Michael E. Kim, Chenyu Gao, Nancy R. Newlin, Gaurav Rudravaram, Aravind R. Krishnan, Karthik Ramadass, Praitayini Kanakaraj, Kurt G. Schilling, Blake E. Dewey, David A. Bennett, Sid O’Bryant, Robert C. Barber, Derek Archer, Timothy J. Hohman, Shunxing Bao, Zhiyuan Li, Bennett A. Landman, Nazirah Mohd Khairi, The Alzheimer’s Disease Neuroimaging Initiative, The HABS-HD Study Team

**Affiliations:** 1 Vanderbilt University, Department of Computer Science, Nashville, Tennessee, United States of America; 2 Vanderbilt University, Department of Electrical and Computer Engineering, Nashville, Tennessee, United States of America; 3 Vanderbilt University Medical Center, Department of Radiology and Radiological Sciences, Nashville, Tennessee, United States of America; 4 Department of Neurology, Johns Hopkins University School of Medicine, Baltimore, Maryland, United States of America; 5 Rush Alzheimer’s Disease Center, Rush University Medical Center, Chicago, Illinois, United States of America; 6 Institute for Translational Research, University of North Texas Health Science Center, Fort Worth, Texas, United States of America; 7 Department of Family Medicine, University of North Texas Health Science Center, Fort Worth, Texas, United States of America; 8 Vanderbilt University Medical Center, Vanderbilt Memory and Alzheimer’s Center, Nashville, Tennessee, United States of America; 9 Vanderbilt University Medical Center, Vanderbilt Genetics Institute, Nashville, Tennessee, United States of America; 10 Vanderbilt University, Department of Biomedical Engineering, Nashville, Tennessee, United States of America; 11 Vanderbilt University Institute of Imaging Science, Nashville, Tennessee, United States of America; University of Minnesota, UNITED STATES OF AMERICA

## Abstract

Thorough quality control (QC) can be time consuming when working with large-scale medical imaging datasets, yet necessary, as poor-quality data can lead to erroneous conclusions or poorly trained machine learning models. Most efforts to reduce data QC time rely on quantitative outlier detection, which cannot capture every instance of algorithm failure. Thus, there is a need to visually inspect every output of data processing pipelines in a scalable manner. We design a QC pipeline that allows for low time cost and effort across a team setting for a large database of diffusion-weighted and structural magnetic resonance images. Our proposed method satisfies the following design criteria: 1.) a consistent way to perform and manage quality control across a team of researchers, 2.) quick visualization of preprocessed data that minimizes the effort and time spent on the QC process without compromising the condition/caliber of the QC, and 3.) a way to aggregate QC results across pipelines and datasets that can be easily shared. In addition to meeting these design criteria, we also provide a comparison experiment of our method to an automated QC method for a T1-weighted dataset of N=1560 images and an inter-rater variability experiment for several processing pipelines. The experiments show mostly high agreement among raters and slight differences with the automated QC method. While researchers must spend time on robust visual QC of data, there are mechanisms by which the process can be streamlined and efficient.

## Introduction

The rate of medical imaging data availability for research is ever increasing as we continue to integrate technology into the healthcare system [1–4]. This influx of imaging data, especially magnetic resonance imaging (MRI) in the field of neuroimaging [5–9], allows us to perform large-scale data analyses important for drawing generalizable and reproducible conclusions about human health [10–13] or more robustly training deep learning algorithms [14]. However, researchers often do not run analyses on these unprocessed image data directly; rather, image processing pipelines are used to extract derived quantitative metrics for subsequent scientific investigations [15]. The scope of derived data can range from first-order features such as total brain volume [16] to complex 3D representations of white matter brain pathways [17]. Unfortunately, not all these available derived data are suitable for use in scientific research, as the algorithmic outputs may not exactly match the features these pipelines are attempting to capture for any given input scan [18]. Implementing robust quality control (QC) protocols can help ensure the quality and consistency of data used for research, facilitating more accurate and reproducible scientific breakthroughs [19] and better machine learning models [20].

There are several considerations for ensuring effective QC of large-scale datasets. First, as neuroimaging datatypes, such as diffusion-weighted imaging (DWI), have multiple preprocessing steps before arriving at the desired downstream outputs, there are several steps that must be checked for quality. As a result, it quickly becomes infeasible for a single researcher to perform all QC tasks as more data are introduced; thus, QC can become more achievable when it is partitioned to members of the team that process the data. However, there are additional considerations for a team-driven effort, such as the manner in which QC for a pipeline is performed. There are a multitude of freely downloadable image viewers available for researchers, and while most provide the same general functionality, each is usually specialized for a specific data type. For example, FSLeyes [21] is specialized for viewing timeseries data and outputs from FSL [22] processing, whereas MI-brain [23] is built for visualizing tractography, but both are capable of viewing 4D imaging data in similar ways with differences in presentation to the user and the graphical user interface (GUI). Moreover, such types of image navigation with viewers for QC are completely unconstrained, further increasing the variability in the QC process, and each individual’s own personal method of image navigation is difficult to communicate to others and reproduce. Any such discrepancies in any aspect of the QC process can greatly increase the amount of time spent aggregating results across a team or can result in data being assessed with different standards. Thus, the remaining time available to perform analyses on the data can be greatly impacted and can even result in data of different quality standards being introduced in the dataset, which can impact results and conclusions from scientific studies [19,24–26]. One innovative method for multi-user QC is an approach like the MRIQC Web-API [27], which aims to crowdsource quality ratings of data submitted by users of the tool. However, crowdsourcing can be problematic, as it relies on a diverse set of raters who may have varying levels of expertise and differing opinions, leading to inconsistent results for contested data. Additionally, for in-house pipelines that are not widely used by researchers, such crowdsourcing methods are not a viable option due to the limited pool of contributors.

Further challenges related to the aspect of team QC are how the QC status of data is being reported and the standards by which researchers assess poor or good quality. While a more complex QC method that assesses multiple dimensions of quality or indicates presence of specific artifacts can provide more insight into the data [28–30], such an assessment would greatly increase the amount of time spent performing QC. For example, a metric of quality such as “accuracy” as defined by Kindling et al., Goasdoue et al., and Taleb et al. [29,31,28], could be used in a deeper dive for what makes certain qualities of data more susceptible to algorithm failure. However, the foremost goal of pipeline QC when handling large-scale MRI data should not be complex judgement of data quality, especially for image processing researchers whose area of expertise does not pertain to MRI scanner systems and software that collect data. No image processing algorithm is perfection incarnate, especially not deep learning-based processing pipelines [18]. Thus, in QC of this scale, the most important aspect is to assess if the algorithm performed as expected or if it failed to produce a believable output.

To that end, another critical concern is the amount of time spent actually performing QC on the data. Though QC is essential for any research study, it is beneficial to optimize the amount of time spent on the process. As mentioned above, there is a panoply of visualization tools available for looking at data. However, opening individual files in these image viewers is far too time-consuming. Time minimization of QC is a well-researched area in imaging informatics. MRIQC and other works have focused on fully automated or semi-automated outlier detection using image-derived metrics [9,32,33]. Semi-automated methods such as MRIQC [32] and ExploreQC [9] provide interactive reports of the image-derived metrics for datasets of minimally processed or unprocessed MRI, with the functionality to select individual outlier scans to perform additional manual QC through a visual report. Fully automated QC methods, such as the method described in Alfaro-Almagro et al. for UK Biobank [33] and the automated classifier released alongside MRIQC [32], use image-derived metrics to exclude images without any visualization. There have also been efforts to automate QC of outputs from processing pipelines such as segmentation algorithms [34,35]. While such methods and tools can considerably cut down the time spent on QC, they are techniques for batch QC of data reliant on quantitative summary metrics rather than QC of individual images and outputs. These batch QC methods cannot consistently capture when an algorithm breaks or fails because they rely on summary metrics of the data that could be unrepresentative of the true data quality: one cannot correctly represent quality of a high-dimensional signal such as a 3D MRI using a single, derived quantitative value. Additionally, an algorithm can work as expected, but still flag data as an outlier due to the nature of the original data, even when the quality is good. Although these “false negatives”, or good quality data flagged as problematic, can be caught with semi-automated approaches to QC, “false positives”, or poor quality data that are not flagged, can still slip through the cracks.

Thus, proper QC requires visualization of every image or data output to ensure that the algorithm performed as expected while remaining efficient with regard to the amount of time spent. In addition to the research on reducing the time spent on manual QC, there exist many tools that are built to provide ease of image inspection so that every output can be visualized. Benhajali et. al proposed a method for quick QC of image registration that involved visualizing each result but was limited only to image registration pipelines [36]. The FSL tool *slicesdir* [22] allows for bulk QC by creating an HTML with orthogonal slices of several NIFTI files for visual inspection but cannot overlay processing such as segmentations. Moreover, *slicesdir* does not provide functionality for nicely aggregated QC results. *snaprate*, a QC tool built to work with XNAT databases, provides more flexibility for QC of pipelines, but does not allow for separation or aggregation of QC results across pipelines [37]. The comprehensive visual reports provided by tools such as MRIQC [32] and fMRIPrep [38] provide a great level of depth in visualizing image data and explaining the steps of their respective workflows; however, such extensive reports are time-inefficient when QC must be performed on a larger-scale and are limited to specific processing and modalities. Some pipelines, such as PreQual [39], already create QC documents designed to assess the quality of specific outputs. However, most times these pre-existing QC documents, like fMRIPrep and MRIQC, are not in a form that is optimized for high throughput quality checks.

**Fig 1:**
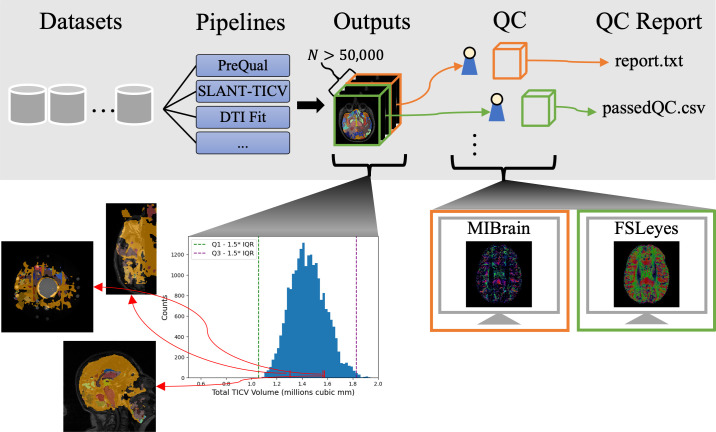
Issues with automatic and team-based QC. When maintaining large neuroimaging datasets with multiple processing pipelines, shallow quality control processes that rely on derived metrics can fail to catch instances of algorithmic failures. However, deep QC processes quickly become unscalable and inefficient as the amount of data available increases due to the required time for mass visualization of outputs. For example, opening 50,000 T1w images separately in an image viewer for deep QC can take over 60 hours if it takes five seconds to load images in and out of the viewer. Team driven efforts to alleviate such large time costs come with additional challenges due to inconsistencies in reporting and methods of performing QC.

To address the above concerns (Fig 1), we propose that an effective QC process for team-driven, large-scale neuroimaging datasets should meet the following design criteria: 1.) a consistent way to visualize the outputs of each image processing pipeline that is run on the data, 2.) a method for quick visualization and QC assessment in order to minimize the amount of time spent on the QC process without compromising the integrity of the QC process, and 3.) a manner by which QC results from different researchers can be easily and seamlessly aggregated across datasets and pipelines to provide a comprehensive QC of the entire database. Addressing these criteria in a QC pipeline can help ensure brevity and completeness in QC while providing a coherency of global QC across all datasets.

**Fig 2:**
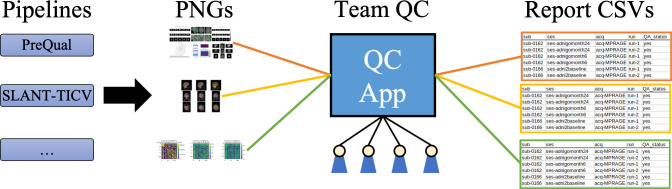
Proposed pipeline for efficient manual QC. We propose an efficient and scalable method for standardized deep QC of neuroimaging pipeline outputs that works within a team setting. For each pipeline output, we provide a visualization as a PNG file. The PNGs are then loaded into a QC app that permits quick scanning of outputs for abnormalities and failures. Any team member can start an instance of the app, and results are standardized in a structured CSV file for each pipeline.

We propose a QC system for a database consisting of national-scale diffusion-weighted imaging (DWI) and T1-weighted (T1w) MRI that adheres to the aforementioned design criteria. Our database consists of 20 large-scale datasets with 16 distinct preprocessing and postprocessing pipelines. We visualize all outputs in the portable network graphics (PNG) format. For viewing the PNGs and assessing quality, we build an application using a Flask (https://github.com/pallets/flask) server that allows researchers to quickly look through PNGs and easily document when data processing has failed (Fig 2). The documentation method also allows us to easily aggregate QC assessments across pipelines and datasets in a CSV format that can easily be shared with collaborators or other team members. Further, we compare our pipeline with an existing method for QC of T1w MRI, *mriqc-learn*, and assess the variability of raters for several different processing pipelines using four different datasets.

## Methods

Upon receiving MRI data, we convert from the Digital Imaging and Communications in Medicine (DICOM) [40] to Neuroimaging Informatics Technology Initiative (NIFTI) (https://nifti.nimh.nih.gov/nifti-1) format when data are provided as DICOM files; no conversion is required if data are only provided in the NIFTI format. For an initial rapid filtering of data, we initially ignore any DWI that do not have corresponding BVAL and BVEC files that indicate the direction and strength of the diffusion-weighting, asking data providers for missing files if possible. We ignore DWI with fewer than 6 volumes unless it is a reverse-phase encoding scan for susceptibility distortion correction that accompanies a more highly sampled DWI. We also visualize raw T1w and DWI in batches using *nibabel* [41] to load images and *matplotlib* [42] to display and output them as PNGs to ensure that scans are the correct modality. We organize raw and derived data according to the Brain Imaging Data Structure (BIDS) (v.1.9.0) format as mentioned in our previous work [43].

### Data processing pipelines

**Fig 3:**
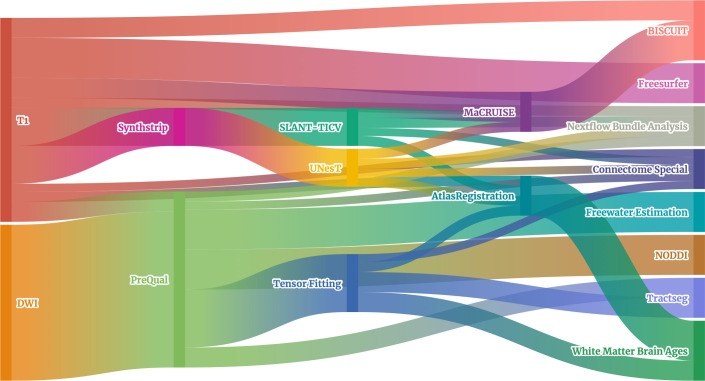
Processing pipeline dependency chain. The 16 pipelines we run on our data form a complex dependency chain, and thus, it is essential to perform quality control on each step so that data are not misinterpreted during downstream analyses. Created with *flourish.studio.*

For processing raw MRI scans, we run 16 different pipelines in total across T1w and DWI modalities (Table 1). The pipelines have a variety of purposes, including preprocessing, signal modeling, image registration, segmentation, tractography, connectomics, and surface reconstruction. There exists a dependency chain for the pipelines, with many also requiring both T1w and DWI modalities (Fig 3).

**Table 1:** Pipeline list. A list of all the processing pipelines that are run on our datasets.

Pipeline	Modality	Description
PreQual [39]	DWI	A preprocessing pipeline for DWI data that includes denoising and correction for motion, susceptibility-induced, and eddy-current induced artifacts as well as slice-wise signal dropout imputation. If multiple images are provided, inter-scan normalization is performed as well. May require a T1w image if no reverse phase encoding scans are available. Also outputs a PDF for quality checks.
Tensor Fitting [44]	DWI	Uses MRtrix3 algorithms to model the diffusion signal as a tensor from preprocessed DWI data (*dwi2tensor*) with volumes <= 1500 and then subsequently extract tensor feature maps for FA, MD, AD, and RD (*tensor2metric*).
EVE3 Atlas Registration [45–47]	DWI/T1w	Using ANTs SyN registration, aligns the JHU template to a preprocessed DWI scan. Also calculates the calculate the mean, median, and standard deviation of the tensor scalar maps for each region in the EVE3 atlas. Requires a T1w image from the same scanning session and uses SLANT-TICV or UNesT segmentation for a brain mask. (https://github.com/MASILab/AtlasToDiffusionReg.git)
MNI152 Atlas Registration [45,48]	DWI/T1w	Affine and deformable registration of the preprocessed DWI to the MNI 152 template using ANTs SyN. Requires a T1w image from the same scanning session and uses SLANT-TICV or UNesT segmentation for a brain mask. (https://github.com/MASILab/AtlasToDiffusionReg.git)
NODDI [49]	DWI	Uses a scilpy script to fit the NODDI model to preprocessed DWI data and export scalar maps of the tissue parameters for the model. Only applicable to multi-shell DWI data. (https://github.com/scilus/scilpy)
Nextflow Bundle Analysis [50–58]	DWI/T1w	A fusion of four separate pipelines using Nextflow: Tractoflow, RecobundlesX, Tractometry Flow and Connectoflow. Nextflow generates whole-brain tractography, RecobundlesX is a multi-atlas segmentation of white matter bundles, Tractometry Flow segments whole-brain tractography into 36 bundles and calculates average tensor scalar values within entire bundles and subsections, and Connectoflow generates connectomics matrices. Requires a T1w image from the same scanning session and uses SLANT-TICV or UNesT segmentation.
Connectome Special [44,59]	DWI/T1w	Constructs a structural connectome of the brain and derived complex graph measures from preprocessed DWI using a combination of MRtrix3 tools and *scilpy* scripts. Requires a T1w image from the same scanning session and uses SLANT-TICV or UNesT segmentation.
Tractseg [17]	DWI	Segments 72 unique white matter bundles using the Tractseg pipeline. Taking the Tractseg bundles, then uses *scilpy* scripts to calculate the mean and standard deviation of tensor scalars within each bundle-defined region of interest.
Free water Estimation [60]	DWI	Uses the Pasternak et al. method of free water estimation and extraction from a diffusion tensor model.
White Matter Brain Ages [61,62]	DWI	Gives estimates of the white matter age from FA and MD scalar maps in the MNI space both with and without minimizing the use of macrostructural information.
SLANT-TICV [16,63]	T1w	A deep learning-based segmentation of the brain into 133 labels under the BrainCOLOR protocol in addition to two labels for the intracranial vault and posterior fossa.
SynthStrip [64]	T1w	Creates a brain mask from a T1w image. Only used when T1w images are defaced, as defacing methods impact results of segmentation algorithms that expect a non-altered T1w image.
UNesT (Yu et al., 2023, 2024)	T1w	Used for BrainCOLOR parcellation of a skull-stripped T1w image, as a separate model was trained for segmentation of skull-stripped images.
MaCRUISE [65]	T1w	Refinement of the BrainCOLOR labels from SLANT-TICV or UNesT based on a cortical surface reconstruction of the brain.
BISCUIT [16,66,67]	T1w	Performs cortical surface reconstruction and estimates curvature, folding, and shape measurements.
FreeSurfer [68]	T1w	Uses the recon-all tool to perform cortical reconstruction of a T1w image with the FreeSurfer toolkit.

### Generation of quality control files

The format we select for viewing outputs is PNG, since Joint Photographic Experts Group (JPEG) format is a lossy compression that does not retain all image information whereas portable document format (PDF) files can consist of multiple pages and have a large file size, making them slower to load. Some pipelines require generation of PNGs from outputs, whereas the atlas registration pipelines and the Connectome Special pipeline output a QC PNG that can directly be used in our process. PreQual [39], the Brain Shape Computing Toolbox (BISCUIT) [66,67,69], and Nextflow Bundle Analysis (https://github.com/MASILab/francois_special_spider.git) output multi-page QC PDFs as part of their respective pipelines, and so we combine these pages into a single PNG for more efficient QC. We automatically determine a process to be a failure for pipelines that do not produce all of their intended outputs, including the QC PDFs/PNGs when applicable.

For the remaining pipelines that require generation of a QC PNG, we use a variety of Python libraries to load and view images in a way that best allows us to perform efficient QC. We combine tensor fitting and atlas registration as a single QC step, overlaying the atlas label map on a fractional anisotropy (FA) map that is extracted from the DWI tensor fit using *nibabel* and *matplotlib* for a tri-planar view at three slices. We similarly overlay the segmentation outputs from UNesT [70,71], SLANT-TICV [16], and MaCRUISE [65] over the T1w image used as input. The free water estimation pipeline QC step has side-by-side triplanar views of the uncorrected and free water corrected FA map in three slices. To visualize each of the individual bundles for Tractseg [17], we use *scilpy* (https://github.com/scilus/scilpy) to overlay the bundles on tri-planar views of the FA map. For Synthstrip [64], we simply visualize the skull-stripped T1w image for all three planes. For NODDI [49], we visualize all tissue parameter maps in three axial slices. Finally, the *fsqc toolbox* (https://github.com/Deep-MI/fsqc.git) was used to generate QC PNGs for cortical surface segmentations of the brain. All PNG files are organized separately from the raw and processed data in a QC archive.

When appropriate, we also include summary quantitative information along with the visualizations. For NODDI outputs or tensor-based images, we include color bars to help interpret the range of values present in the scalar maps. PNGs for segmentations and parcellations, such as SLANT-TICV and FreeSurfer, have global tissue volumes such as whole brain volume, white matter volume, gray matter volume, etc. When visualizing TractSeg tracts, we include the number of reconstructed streamlines to help raters infer tract density for instances where a tract appears fully reconstructed but has a small streamline count. As a structural connectome is often used to calculate derived graph measures, we include many representative metrics, such as assortativity, rich club coefficient, etc.

### Performing quality control for a dataset and pipeline

**Fig 4:**
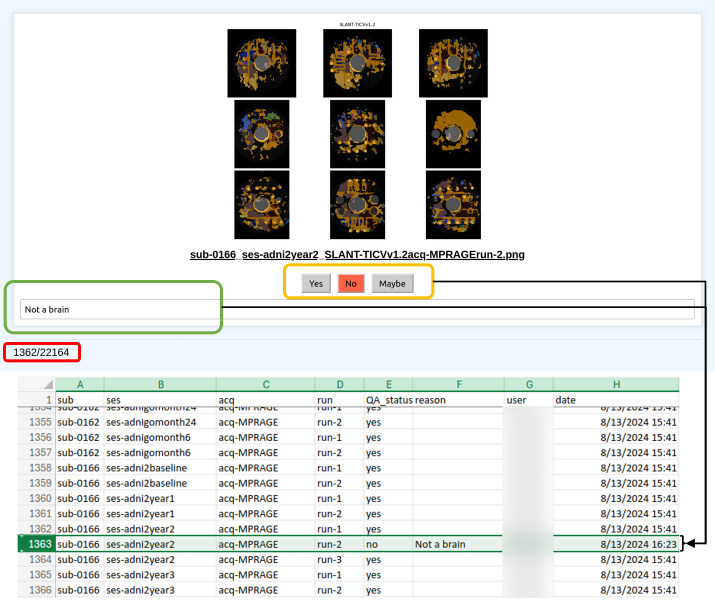
QC app interface. The proposed QC app enforces consistent visualization of pipelines outputs through uniformly generated QC PNG documents for each respective pipeline. The homogenous format of the documents and high success rate for pipelines allow QC users to very quickly cycle through PNGs to catch any abnormalities. Moving through PNG documents can be done either manually with the arrow keys or automatically through the montage feature of the app (not shown). A counter is maintained (red box) so the user can know how many documents are left to view. Most outputs are expected to be good, and thus all QC results are initialized as “Yes” (passes QC) in order to minimize the manual effort in reporting. If the output is not satisfactory, the user can click the corresponding button to indicate their decision (yellow box) and articulate the reason for their verdict with some accompanying text (green box). Any changes are automatically pushed to a CSV document that maintains structured information about the results of the QC for a pipeline and dataset. All QA CSV results documents are formatted the same way and can thus be easily merged across pipelines and datasets so that QC decisions can be shared among team members and collaborators in an easily parseable format.

**Fig 5:**
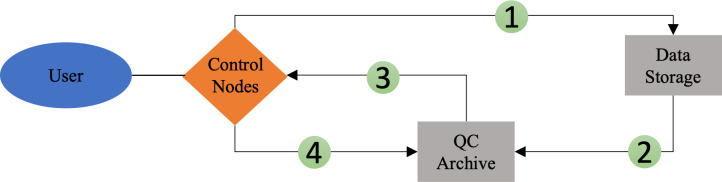
Diagram for QC workflow. Our method allows for an efficient quality control pipeline: 1.) QC files are generated by querying the data storage from control nodes 2.) and then stored on an archive separate from the data. 3.) To perform QC, files are pulled from the QC archive 4.) and then QC results are pushed back in real time for other researchers to use.

Once we have generated the PNG files for a pipeline that has been run on a dataset, we visually inspect each file using a QC app running on a Python backend with Flask (Fig 4). Each researcher can start their own instance of the QC app and select the dataset and pipeline that they wish to perform QC on. PNG files are then pulled from the QC database and loaded in the QC app for the user to view. Whenever a failed pipeline is detected, the user can document that the output is a failure, and the results will be pushed in real-time to a database of the QC results for that pipeline (Fig 5). The source code for the app can be found here, in a publicly available repository: (https://github.com/MASILab/ADSP_AutoQA.git). We also make a BIDS-agnostic version available to download as well for researchers who cannot adhere to the BIDS organizational standard, but still wish to perform QC using our app: (https://github.com/MASILab/GeneralQATool).

### Adherence to design criteria

#### Consistent visualization.

In order to ensure a single consistent visualization practice across all researchers, we perform all QC of pipeline outputs using the Python QC app, with all QC files having the same PNG image format. Thus, there is no variety in the manner in which outputs are viewed, preventing any preferences for tools or image viewers from causing variations in the QC process. Further, we use the same method for each pipeline to create QC PNGs across all datasets, enforcing all members of the QC team to visualize each respective pipeline output in the same way. This method also makes expectations of proper pipeline outputs homogenous across our QC team.

#### Efficient and proper quality control minimizing time and effort.

As visually looking at every pipeline output is of utmost importance for a deep QC process, we ensure that all outputs are viewed by a QC team member. As mentioned in Section 2.2, the PNG format is a single image, allowing for fast loading times and easier visualization unlike PDFs, while still maintaining a high image quality, unlike lossy compression formats such as JPEG. The aspect of the QC process that best increases time efficiency without sacrificing quality of QC is the Python QC app, which permits users to quickly cycle through PNGs for an entire pipeline of a dataset. The app pre-loads the images into memory prior to visualization, permitting incredibly fast refresh speeds on the user’s monitor. To further reduce the effort of the QC process, the app allows the user to automatically montage the PNGs at either a faster or slower speed depending on the preference. If there is an output that the user finds issue with, they may stop the montage at any time to record a failed QC instance. The user also has freedom to cycle back and forth between images in case they would like to return to a previously viewed output.

Another point of efficiency is the minimal effort of reporting QC results. As we expect most outputs to be properly processed data, the status of all outputs is initially set to have passed QC, minimizing the amount of manual effort in reporting. If there is a bad output encountered, reporting the status requires only clicking the button that indicates a failed output. If further explanation is needed, there is also the option of entering a small text blurb to accompany the QC decision. Finally, after the user has reported the QC status, any detected change is automatically pushed to the archive for other users to see.

#### Consistent reporting and aggregation of results.

To ensure a uniform QC reporting format, the QC app permits users to report status as one of three options. “Yes,” means that the pipeline has properly run on the input data. “No,” means that there is a failed instance of the algorithm in some manner, where the output is an unexpected result. Lastly, “Maybe,” is a more nuanced decision that indicates that the algorithm performance is within expectation or believable, but the outputs are not visually good results. For example, a “Maybe” instance could be if a tractography algorithm produced a very small number of streamlines for a white matter bundle that are anatomically correct, but the bundle is not as thick as it could be. A “Maybe” is intended to leave the decision up to the researcher who is using the data for their analysis. Should there be any additional need for clarification, the user may also add some text to explain their QC decision. Thus, there is no ambiguity and little subjectivity in the reporting of results, as opposed to methods such as a rating scale.

For each dataset and pipeline, the QC results are also stored in a consistent format as a CSV file, with a row for each individual output for a pipeline run on the dataset. The columns indicate identifiers, such as the BIDS tags for the corresponding data, as well as all the QC information, including the QC status, user, time of the QC report, and any notes left by the QC reporter. As all QC reports are stored in this consistent CSV format, the results are simply aggregated together with a single script to create a QC document that encompasses the results across all pipelines of a dataset. The CSV is easily readable and can be shared with any other team members or collaborators who use the data.

### Experimental

To assess our QC pipeline, we compare to an existing method for automated QC. We also assess inter-rater variability of the QC method using outputs from several pipelines that we currently run on our database. In both our comparison experiment and our inter-rater variability experiment, we time how long it takes to perform QC using the QC app. We use a subset of our maintained datasets for the experiments.

#### Comparison to MRIQC classifier.

We use *mriqc-learn*, an automated classifier trained as described in Esteban et al. [32] to classify T1w images as pass or fail based on the 64 image quality metrics output by the MRIQC pipeline. [72] To run MRIQC, we download the latest containerized version (version number 24.1.0) from Docker hub at (nipreps/mriqc) as a singularity image. For the data used in the comparison experiment, we select N=1561 T1w images from the Wisconsin Registry for Alzheimer’s Prevention (WRAP) dataset. WRAP, begun in 2001, is a study of midlife adults enriched with persons with a parental history of AD [73]. We have one rater perform QC using our QC app to classify images based on the image quality (See Supplementary Information Section A for the prompt provided to the user performing QC). The QC PNGs generated for the T1w images are made using *nibabel* and *matplotlib* for a tri-planar view at three different slices. We then compare the agreement between the two methods to assess the differences in QC performance. Timing information of how long the QC app method takes is also collected by the rater performing the QC.

#### Assessment of QC app inter-rater variability.

To compare how different the variability in QC is when using our QC app, we have four raters familiar with DWI and T1w images, one of which participated in the MRIQC classifier comparison experiment, perform QC on multiple pipeline outputs. Specifically, we have the raters QC outputs from PreQual (N=315), SLANT-TICV (N=1000), and five different tracts from TractSeg (N=1000 each): the right arcuate fasciculus (AF right), the anterior midbody of the corpus callosum (CC4), the left corticospinal tract (CST left), the right thalamo-postcentral right (TPOST right), and the left superior longitudinal fascicle I (SLFI left). For the pipeline outputs included in the experiments, we use all data that were previously rejected in a prior assessment due to poor quality and then randomly select from the non-rejected samples until the predetermined sample size is reached (see Supplementary Information Section C: Table SC.T1 for more details). This is done to ensure that data of a questionable quality were included in the experiments. Data used for PreQual and TractSeg processing come from the Health and Aging Brain Study – Health Disparities (HABS-HD) dataset, whereas data used for SLANT-TICV processing come from the Alzheimer’s Disease Neuroimaging Initiative (ADNI). ADNI (www.adni.loni.usc.edu), begun in 2003, was designed to assess brain structure and function using serial MRI, other biological markers, and clinical and neuropsychological assessments [5]. Three ADNI phases (ADNI-GO, ADNI 2, and ADNI 3) are included in the present study. The HABS-HD project (previously the HABLE study) seeks to understand the biological, social, and environmental factors that impact brain aging among diverse communities and is dedicated to understanding and eliminating health disparities in Alzheimer’s disease among African Americans and Mexican Americans [74]. We provide the same set of instructions to each rater on how the QC should be performed (See Supplementary Information Section A for the prompts provided to the users performing QC). The results from each rater are then compared for agreeability among raters quantitatively using Fleiss’ Kappa [75]. Similar to the MRIQC classifier comparison experiment, each rater timed themselves for each individual QC task.

To demonstrate the generalizability of the QC tool outside of the neuroimaging MRI community, we perform another inter-rater variability experiment assessing quality of outputs from a deep learning lung computed tomography (CT) harmonization pipeline. Specifically, we select N=480 CT images of the chest that are the result of kernel harmonization using the method from Krishnan et al. [76], where CT images from GE BONE and Phillips C kernels (N=240 randomly selected from each) are harmonized to a Siemens B30f kernel. Data used for the experiment come from the National Lung Screening Trial (NLST) [77]. We have provided the instructions sent to reviewers for the QC experiments in Supplementary Information Section A.

## Results

**Fig 6:**
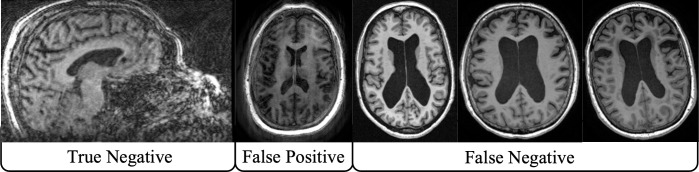
Qualitative results for MRIQC classifier comparison experiment. Moderate to severe motion artifacts can be seen in the scan rejected for both methods (“True Negative”) and the scan deemed acceptable by MRIQC but rejected by the reviewer using the QC app (“False Positive”). Scans that were rejected by MRIQC but passed by the reviewer (“False Negative”) had qualities typical of older brains, such as larger ventricles, despite being of acceptable quality.

When running MRIQC on all data, only one scan was unable to run through MRIQC without early termination. As there were no image quality metrics generated, this single scan was removed from the analysis (N=1560). Ratings given to the T1w images were mostly positive for both the MRIQC classifier and the rater using the QC app, with only a small number of rejections from both methods. The agreement between the two methods was also very high, as seen in the resulting confusion matrix (Table 2). For the purpose of brevity, we use “false negative” to indicate a scan that received a passable rating by the rater using the QC app but was rejected from the classifier, and “false positive” for a scan that was rejected by the rater but not rejected by the classifier. Only two scans were rejected from the rater whereas five were rejected by the classifier, with four of those being false negatives. When visualizing all rejected scans, the false negatives all had qualities typical of aging brains, such as large ventricles, whereas the one false positive and “true negative” (when both methods reject the scan) both had moderate to high motion artifacts (Fig 6). The recorded time for the rater using the QC app was 10 minutes and 36 seconds.

**Table 2:** Confusion matrix for MRIQC classifier experiment. A confusion matrix indicates that agreement between the QC app and the MRIQC classifier for T1w images was high with a few exceptions. We note that one scan failed to run through MRIQC and thus could not be included in this analysis.

	MRIQC Pass	MRIQC Reject	Total (*Time = 10:36*)
QC App Pass	*1554*	*4*	**1558**
QC App Reject	*1*	*1*	**2**
**Total**	**1555**	**5**	**1560**

**Fig 7:**
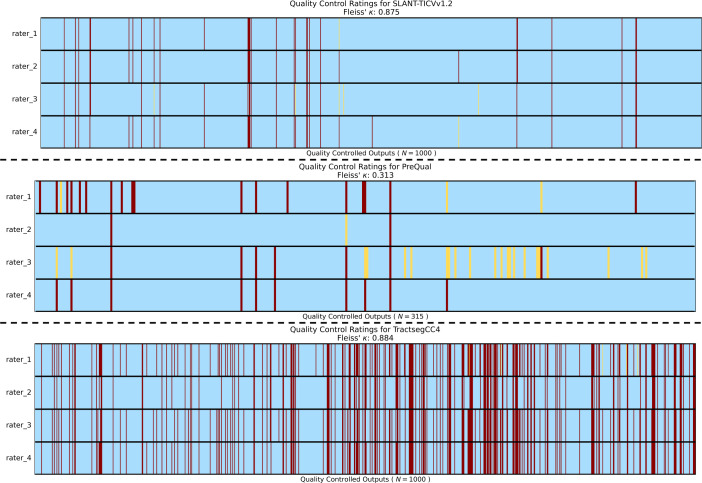
Inter-rater variability results for PreQual, SLANT-TICV, and the CC4 tract. Inter-rater variability of the four raters for QC of both SLANT-TICV (top) and TractSeg tract CC4 (bottom) using the QC app showed very high agreement, with Fleiss’ Kappa values greater than 0.8. The agreement was fair for PreQual QC (middle) with a Fleiss’ Kappa in the range 0.2–0.4. Blue indicates that the rater selected “yes” for the output while yellow and red indicate selection of “maybe” and “no” respectively.

**Fig 8:**
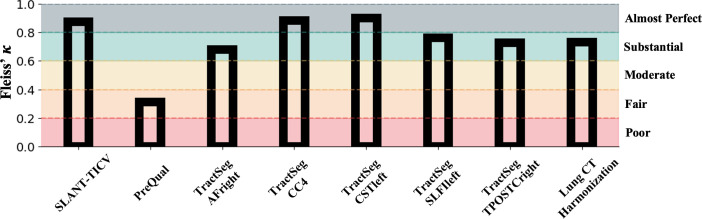
Fleiss’ Kappa scores for QC inter-rater variability experiments. Fleiss’ Kappa agreement scores for QC pipelines across four raters suggest high inter-rater variability. Different levels of agreement as presented in Landis and Koch20 are shaded in with labels to the right of the plot. SLANT-TICV, TractSeg CC4, and TractSeg CSTleft are QCed with “almost perfect” agreement, whereas TractSeg AF right, TractSeg SLFIleft, TractSeg TPOSTCright, and Lung CT Harmonization have “substantial” agreement. PreQual is the only pipeline QCed with “fair” agreement.

The inter-rater variability among the four was either substantial or almost perfect agreement for most pipelines. According to Landis and Koch, agreement among raters is “fair” when the Fleiss’ Kappa value is between 0.21 and 0.4, “moderate” for 0.41 to 0.6, “substantial” for 0.61 to 0.8, and “almost perfect” when greater than 0.8 [78]. The only pipeline not at least at a “substantial” level of agreement was PreQual, which had a Fleiss’ Kappa of 0.313, whereas agreement for the Lung CT Harmonization and TPOSTC right, SLFI left, and AF right tracts fell into the substantial category and SLANT-TICV, CST left, and CC4 fell into the almost perfect category (Fig 7, Fig 8, Supplementary Figure Section C: Fig SC in S1 File). We do note that the amount of information present for each pipeline specific QC PNG is different, which may affect the variability of raters. Timing information of all raters for each pipeline can be found in Supplementary Information Section B: Table ST.B1. Additionally, examples of pipeline outputs can be found in Supplementary Information Section C: Figs SC.F2 and SC.F3 in S1 File.

## Discussion

For a comprehensive QC of pipeline outputs, visual inspection of every output is paramount to ensure that no instances of failed algorithms are used in scientific analyses. As dataset size and the number of processes scale up, multiple researchers are needed to perform QC in a timely manner. We have demonstrated a proposed method of facilitating visual inspection that permits consistent and efficient QC across a team setting. The use of a unified QC app that enforces reporting of results in a specific manner promotes stability of the team QC. Additionally, the QC PNGs are generated in the same way for each individual pipeline. The expectation of what a proper pipeline output should be, coupled with the consistency in format of the output PNGs, increases efficiency by permitting rapid cycling through the QC PNG documents with the QC app. The standardized visualization of outputs can also help reduce the amount of training required for new team members who have little to no experience with QC of the pipelines. Further, our automated documentation of QC results to the QC archive and ease in aggregating QC across pipelines reduces the manual effort for compiling the efforts made across the entire team. Combining results in an easily parseable CSV format also helps to expedite research and analysis for team members who use the data and for external collaborators. In these ways, we have successfully met the design criteria for a deep QC pipeline. We have also demonstrated in our QC experiment for the Lung CT Harmonization pipeline that this QC tool can be applied to fields outside of the neuroimaging domain.

In previous work, researchers have proposed methods for visual QC of outputs. The Mindcontrol QC application developed by Keshevan et al. links visualization of outputs with quantitative metrics and manual annotation tools [79]. However, Mindcontrol is not suited for visualizing large volumes of data as it loads in entire 3D medical images in order to allow users to annotate and modify errors in the processing pipelines. The MRIQC and fMRIPrep QC tools create QC visualizations without loading in entire 3D volumes, but their visualization methods prevent quick QC as every single axial slice is rendered in the QC image [32,38]. Alternative solutions that permit rapid visualization, such as FSL’s *slicesdir*, do not allow flexibility in how visualizations can be structured [22]. Our QC tool is unique in that it provides a method for rapid visualization of outputs in a pipeline agnostic manner.

For our visualizations of each pipeline presented in Table 1, we build off established methods from other researchers. With regard to deep learning segmentation, Benhajali et al. posit that triple slice, tri-planar visualizations of label maps overlayed on a structural scan is best for rapid QC [36]. As segmentation of white matter regions is the ultimate task for our atlas-based registration pipelines, we have found that overlaying the registered label maps in a similar manner is effective for efficient QC of registration. In comparing their visualization method to others, Raamana et al. suggest that contour maps and comprehensive views of surface-based parcellations result in more reliable QC between raters [80]. Following their example, we ensure visualizations of the BISCUIT and FreeSurfer pipeline outputs contain these as well. For bundle-specific tractography QC, He et al. provide a semi-automated method with manual editing of erroneous outputs [81]. However, manual editing-based methods like Mindcontrol or that in He et al. are not appropriate for efficient QC of a large volume of data. Still, He et al find that overlaying white matter bundles on an FA map is appropriate for identifying erroneous outputs. Thus, we combine this concept with the tri-planar viewing method to allow for both efficient and appropriate QC of TractSeg outputs. For pipelines with larger QC documents included as part of the outputs, such as PreQual [39], Nextflow bundle analysis, and BISCUIT toolbox, we maintain as much information in the original documents as possible that permits us to perform efficient, yet effective, QC. As structural connectomes are two-dimensional datatypes whose size is limited to the number of brain regions of interest, most visualizations of connectome pipeline outputs consist of the entire connectome [56,82]. Thus, our visualization for connectomics pipelines follows this practice. Note that we have not tested any hypotheses comparing different methods of visualizations across pipelines. We also wish to highlight that, while other alternative pipelines exist, such as FSL’s dtifit instead of MRtrix3’s dwi2tensor for tensor fitting or TRACULA for bundle-specific tractography instead of and TractSeg, we have chosen the specific tools in Table 1 for our purposes. Using any alternatives may result in different outputs, even when the tools are intended to perform very similar tasks. Nevertheless, the QC app can be applicable to any pipeline as long as the output can be visually inspected.

Including data of poor quality can impact results, as shown in Ducharme et al. with regard to cortical thickness trajectories changing from non-linear to linear depending on data quality [19]. Such changes in results are likely be due to additional unexplained variances in images being introduced into the dataset, making it more difficult to capture or identify underlying trends in the data as it would require a larger sample size. The inclusion of low-quality data can pose significant challenges for machine learning and deep learning models, as these models are highly sensitive and may learn incorrect data distributions [83]. For FreeSurfer outputs, Monereo-Sánchez et al. found that the largest decrease in unexplained variance occurred when manual QC was performed as opposed to automated QC methods [84]. We hope that the tool presented here will help promote scientific discoveries and better-trained deep learning models by removing unwanted variability due to poor data quality.

Although we advocate for rapid visualization of every image and pipeline output when faced with massive datasets in order to perform a comprehensive QC process, we are not disparaging alternative tools or methods that are slower due to being more comprehensive, such as the individual visual reports generated by MRIQC. Rather, we caution researchers against using these tools in a semi-automated manner where not every output or image is visually inspected and suggest a high degree of caution for fully automated methods. Scenarios of false negatives can be easily remedied for semi-automated methods where visualization occurs only for rejected scans, but in cases of false positives poor quality data can end up in analyses. For example, in our experiment comparing the automated MRIQC classifier to the QC app, one scan out of 1560 was classified as good quality by the classifier but was poor quality when visualized (Fig 6). This low number is in part due to the high quality of the WRAP dataset, where not many scans were qualitatively problematic. Analyses of larger scale, such as the one in Bethlehem et al. [85] that used over 120,000 T1w scans, or datasets of worse overall quality could result in many more scans of bad quality being included and affecting results. Performance of automated or semi-automated classifiers could be improved by training with increased sample sizes and multiple different population distributions (such as different age ranges, patient populations, etc.).

Inter-rater variability was low, with high Fleiss’ Kappa scores for most pipelines (Fig 7, Supplementary Fig in S1 File), even for outputs such as the TPOSTC right tract from TractSeg that had a high number of problematic outputs included in the data sample used for the experiment (Supplementary Table in S1 File). These differences in number of included problematic samples, amount of information in each pipeline’s QC PNG, or the difference in total sample size for the case with PreQual output QC could have influenced the differences in agreement scores across pipelines. Despite our experiments showing mostly low inter-rater variability, there is not perfect agreement. Our results suggest that for team-based QC of pipelines on large-scale datasets, QC of each process should be consistently performed by the same team members, rather than each member conducting QC of all pipelines on a subset of the data. Additionally, we note that the experiments on inter-rater variability were not intended to compare performance across raters. Further, although the four raters for the inter-rater variability experiments all have different levels of experience when working with the datatypes used in the experiments, all come from the same research group. Thus, the variability may change for an alternative set of raters.

One of our main goals in using the PNG format to view outputs was to promote brevity and efficiency in the QC process. We acknowledge that other image formats, such as scalable vector graphics (SVG), exist and could be used as well. However, as efficiency in visualization was our main goal, the PNG format was sufficient for this approach. Furthermore, despite the increased efficiency, we do recognize that visualization with an image viewer that was built to render the datatype being examined would be a more comprehensive level of QC, as a user would have more control in inspecting any part of high-dimensional data rather than a fixed viewpoint. As the QC design criteria were to assess outputs for failure of the algorithm, not a grading of quality level, we believe the decision to draw the line for the efficiency-thoroughness tradeoff should lie closer to the efficiency end. Thus, it was important to briefly visualize the data in a way that would maximize the amount of information in order to prevent caliber of the QC from being compromised. We understand the choice is subjective for what the best ways are to represent the data, and our decisions were based on preliminary QC efforts that we have built upon for the past two years. As we continue to maintain our MRI database, we will look to integrate new and existing tools into our current processing pipeline, such as FastSurfer [86], that will also require QC procedures. We note that the QC tool presented here is agnostic to processing pipelines, whether for newly incorporated tools or those discussed in this manuscript. Specifically, our QC tool provides flexibility to users regarding the content of the images. Researchers can insert visualizations they feel are most appropriate for their QC process, as long as each QC file is in the PNG format. Thus, our tool facilitates the implementation of a standardized QC procedure, which can be customized by each research group to optimize an efficient QC process for large volumes of data. For the rare cases when there is still ambiguity in whether or not the algorithm failed, we do advocate visualization in a proper image viewer to better understand if the output is a successful result. We are not trying to discern why the algorithm may have failed in our pipeline, but if users wish to understand the algorithmic shortcomings, such visualization might help elucidate the issues.

We also note that our QC process is mostly focused on qualitative visualization of outputs rather than quantitative visualization. This is because quantitative visualization on an individual/session level would greatly reduce the efficiency of performing QC, as visualization of images is quicker to investigate than a series of numbers and is often more effective. For instance, a tractogram is a collection of lines in 3D space, where each line (or streamline) is composed of several (x,y,z) coordinates. To quantitatively read the coordinates for millions of streamlines is an infeasible task. Representing the tractogram as a condensed quantitative metric would make the quantitative QC process easier, but then one would be relying on a derived measure instead of visualizing the data itself. As we continue performing QC on our data, we will consider including batch-level quantitative methods of QC on derived metrics, such non-parametric statistical methods like interquartile range (IQR) and median absolute deviation (MAD) for outlier detection [87], in addition to our current pipeline in order to supplement the process. Such additions will be essential for pipelines like the white matter brain age that output only a single value.

Finally, in our approach, we are focused on identification of failed data processing. We do not attempt to correct data beyond any preprocessing that is common in the field for the imaging modality. We also are not trying to perform any iterative algorithmic fine-tuning to produce slightly higher quality outputs (for example, retraining a segmentation algorithm on more data and viewing the outputs for each new model). We suggest researchers who are interested in such algorithmic optimization to use a different method for QC of data, as our method is not intended for grading quality of outputs. We also do not attempt to assess how the quality of outputs from one pipeline influences the performance of a subsequent downstream analysis. Although we opted to use the “yes/maybe/no” categorization for QC ratings, there exist alternative categorizations that could be used instead, such as the one used for the MRIQC Web-API [27] or a Likert scale, which measures a range of ratings and is a frequently used option for grading MRI scan quality [88,89]. Such a rating scale can be beneficial because it allows for image quality to be used as a covariate for downstream analyses [88–90]. However, a recent study from Hoeijmakers et al. found that the rating scales in QC result in decreased reliability and inter-rater agreement when compared to a pairwise comparison (PC) quality assessment, suggesting that the greater availability of rating options reduces consistency [91]. Tang et al. similarly find that PC-like methods result in more reliable scores, even among board certified radiologists, and also observe a significantly faster QC time when compared to rating with a Likert scale [92]. Both Hoejimakers et al. and Tang et al. posit that rating scales increase the amount of subjectivity in QC, even despite having a defined rubric for scale ratings as in Tang et al. As our goal is to decrease subjectivity while increasing efficiency and consistency, we feel having concrete “yes/no” options to indicate processing pipeline failure is an optimal choice to satisfy the design criteria. Further, the “maybe” option allows for more flexibility in cases where a decision is not clear, with the text box provided in the QC app allowing raters to further elaborate on their decisions if necessary. Finally, we note our method is not defined as PC as in Hoejimakers et al. and Tang et al. Such a QC method results in performing $\frac{N(N-1)}{2}$ QC ratings for N outputs as opposed to N ratings when assessing each output individually, which can greatly increase the amount of time performing QC for large datasets as we do. However, the QC app allows raters to easily and quickly return to previously rated PNGs should they need comparison data similar to a PC-based method. In this way, the QC app has the benefits of PC-based methods without incurring the additional overhead in multiple comparisons.

### Code and data availability—information sharing statement

The source code for the QC app can be found here: (https://github.com/MASILab/ADSP_AutoQA.git). A version of the QC app that does not require naming according to the BIDS convention can be found here: (https://github.com/MASILab/GeneralQATool). Note that this second version was not used in preparation of this manuscript, and is provided here out of convenience for users who do not wish to adhere to the BIDS organizational naming and structure.

The datasets supporting the conclusions of this research are available, subject to certain restrictions. The datasets were used under agreement for this study and are therefore not publicly available. The authors may provide data upon receiving reasonable request and with permission.

Data used in the preparation of this article were in part obtained from the Alzheimer’s Disease Neuroimaging Initiative (ADNI) database (adni.loni.usc.edu). The ADNI was launched in 2003 as a public-private partnership, led by Principal Investigator Michael W. Weiner, MD. The primary goal of ADNI has been to test whether serial magnetic resonance imaging (MRI), positron emission tomography (PET), other biological markers, and clinical and neuropsychological assessment can be combined to measure the progression of mild cognitive impairment (MCI) and early Alzheimer’s disease (AD). (https://adni.loni.usc.edu/)

HABS-HD data can be requested from: https://apps.unthsc.edu/itr/our.

WRAP data can be requested from: https://wrap.wisc.edu/data-requests-2/.

## Supporting information

S1 File Supporting information document, File that contains all supporting information (figures, tables, methods) in the manuscript. (DOCX)
