# Supplementary material for: Scalable quality control on processing of large diffusion-weighted and structural magnetic resonance imaging datasets
Source: PLoS One. 2025 Aug 1;20(8):e0327388. doi: 10.1371/journal.pone.0327388 (PMC12316263; doi:10.1371/journal.pone.0327388)
Supplement: S1 File — (DOCX) [file pone.0327388.s001.docx]

SUPPLEMENTARY INFORMATION

Scalable quality control on processing of large diffusion-weighted and structural magnetic resonance imaging datasets

Michael E. Kim^a^*, Chenyu Gao^b^, Nancy R. Newlin^a^, Gaurav Rudravaram^b^, Aravind R. Krishnan^2^, Karthik Ramadass^a,b^, Praitayini Kanakaraj^a^, Kurt G. Schilling^c^, Blake E. Dewey^d^, David A. Bennett^e^, Sid O’Bryant^f,g^, Robert C. Barber^g^, Derek Archer^h,i^, Timothy J. Hohman^h,i^, Shunxing Bao^b^, Zhiyuan Li^b^, Bennett A. Landman^a,b,c,j,k^ , Nazirah Mohd Khairi^b^, The Alzheimer’s Disease Neuroimaging Initiative^A^, The HABS-HD Study Team^B^

^a^Vanderbilt University, Department of Computer Science, Nashville, TN, USA

^b^Vanderbilt University, Department of Electrical and Computer Engineering, Nashville, TN, USA

^c^Vanderbilt University Medical Center, Department of Radiology and Radiological Sciences, Nashville, TN, USA

^d^Department of Neurology, Johns Hopkins University School of Medicine, Baltimore, Maryland, USA

^e^Rush Alzheimer’s Disease Center, Rush University Medical Center, Chicago, IL, USA

^f^Institute for Translational Research, University of North Texas Health Science Center, Fort Worth, Texas, USA

^g^Department of Family Medicine, University of North Texas Health Science Center, Fort Worth, TX, USA

^h^Vanderbilt University Medical Center, Vanderbilt Memory and Alzheimer’s Center, Nashville, TN, USA

^i^Vanderbilt University Medical Center, Vanderbilt Genetics Institute, Nashville, TN, USA

^j^Vanderbilt University, Department of Biomedical Engineering, Nashville, TN, USA

^k^Vanderbilt University Institute of Imaging Science, Nashville, TN, USA

^A^Data used in preparation of this article were obtained from the Alzheimer’s Disease Neuroimaging Initiative (ADNI) database (adni.loni.usc.edu). As such, the investigators within the ADNI contributed to the design and implementation of ADNI and/or provided data but did not participate in analysis or writing of this report. A complete listing of ADNI investigators can be found at: <http://adni.loni.usc.edu/wp-content/uploads/how_to_apply/ADNI_Acknowledgement_List.pdf>

^B^HABS-HD MPIs: Sid E O’Bryant, Kristine Yaffe, Arthur Toga, Robert Rissman, & Leigh Johnson; and the HABS-HD Investigators: Meredith Braskie, Kevin King, James R Hall, Melissa Petersen, Raymond Palmer, Robert Barber, Yonggang Shi, Fan Zhang, Rajesh Nandy, Roderick McColl, David Mason, Bradley Christian, Nicole Phillips, Stephanie Large, Joe Lee, Badri Vardarajan, Monica Rivera Mindt, Amrita Cheema, Lisa Barnes, Mark Mapstone, Annie Cohen, Amy Kind, Ozioma Okonkwo, Raul Vintimilla, Zhengyang Zhou, Michael Donohue, Rema Raman, Matthew Borzage, Michelle Mielke, Beau Ances, Ganesh Babulal, Jorge Llibre-Guerra, Carl Hill and Rocky Vig.

*Corresponding Author: [michael.kim@vanderbilt.edu](mailto:michael.kim@vanderbilt.edu)

**Keywords:** Informatics, MRI, AI Ready, Quality Control

## Supplementary Information Section A: Rater Instructions for Experimental Section

Below are the instructions provided to each rater for the inter-rater variability experiment, sent to raters via email.

*************

The root folder for this QC experiment is located at:

**</path/to/root/folder>**

where all the QA pngs have already been created for you. If you do not already have the QC tool downloaded, please pull it from the following repository (Instructions for setup and using the tool can be found on the github README page):

<https://github.com/MASILab/ADSP_AutoQA>

0.) Please try to access this link: **<*URL_FOR_SHARED_GOOGLE_SHEET*>**. Edit access should have been sent via email. Reply if you do not have access or did not receive the email.

1.) Go to the directory containing the tool and run the command:

python3 app_montage.py **</path/to/root/folder>**

2.) Copy and paste the link into a browser (please use google chrome if possible, as it works best on chrome).

3.) **Have a timer prepared to time how long each process takes.** Click on the folder corresponding to your QC ID.

At this point, you should see a selection of 7 different folders, each corresponding to an MRI processing pipeline (1 for SLANT-TICV, 1 for PreQual, 5 for Tractseg). Clicking on any of them will start up the QC tool that you can use to perform QC on the data. Feel free to QC the pipelines in any order, but please make sure to QC all of them. Below are the ONLY instructions you will receive for how to perform the QC; use your own discretion when making decisions based on these rules and these rules alone:

SLANT-TICV: A "yes" result means that the segmentation algorithm ran as expected and the scan is a full-brain T1w image. A "no" means that the segmentation algorithm failed to produce an expected output or the scan is not a full-brain T1w image.

PreQual: A "yes" result means that the DWI scan is useable in an analysis based on the information in the QC png. A "no" means that EITHER at least one part of the PreQual pipeline failed to work as expected OR the resulting DWI is unusable.

Tractseg: A "yes" means that the bundle produced by the algorithm looks as expected and can be used to obtain reasonable microstructural and macrostructural metrics. A bundle marked "yes" should have a full appearance and be intact. A "no" means that the bundle EITHER is not usable for macrostructural or microstructural analysis OR the bundle failed to reconstruct as expected OR does not have a full/intact appearance.

If you feel that none of the descriptions above match your interpretation for the quality of the output, you may mark the particular output as "maybe". Please feel free to use the provided text box to give any accompanying text for your decision if you feel it is necessary. Once you are finished with QC of a pipeline, please make sure to record the time that it took to finish the QC of the pipeline here in this google sheet: **<*URL_FOR_SHARED_GOOGLE_SHEET>***

4.) Once you have finished all your QC tasks, please reply to this thread with the message: "Complete!"

**********

In addition to these instructions, one rater was provided with the following set of instructions for QC of the T1-weighted images to be used in the experiment comparing to the MRIQC classifier:

**********

In addition to QC of the outputs from the three aforementioned processing pipelines, there is also a folder labeled "T1w", which contains T1-weighted brain images from the WRAP dataset. For QC of these images, please adhere to the following instructions (please time QC of this folder as well):

QC of T1-weighted images should be based on whether or not the scan would be acceptable for use in downstream NEUROIMAGE processing pipelines or analyses. A scan should be marked as "yes" if it is free of any image artifacts or abnormalities that would interfere with downstream quantitative image processing or analysis pipelines. A scan should be marked as "maybe" if there are mild image artifacts or other abnormal aspects of the image present, but the presence of these artifacts/abnormalities likely would not affect downstream quantitative image processing or analysis pipelines for neuroimaging. A scan should be marked as "no" if the quality of the scan is poor to the point that it would be unusable or unreliable in downstream analyses.

While not comprehensive, such artifacts or abnormalities may include "ghosting/motion artifacts", "aliasing/wrap-around", "gibbs ringing", presence of non-biological objects (such as implants or headphones), etc. Further information on some types of image artifacts and their effects can be found in this paper (https://pmc.ncbi.nlm.nih.gov/articles/PMC4340093/), titled: "Artifacts in Magnetic Resonance Imaging."

If you do detect something in the scan that causes you to classify it as a "maybe" or a "no", please mark it down in the text box provided.

**********

We also provide instructions given to raters for QC of the lung computed tomography harmonization pipeline outputs, also sent via an email:

**********

The root folder for this QC experiment is located at:

**</path/to/root/folder>**

where all the QA pngs have already been created for you. If you do not already have the QC tool downloaded, please pull it from the following repository (Instructions for setup and using the tool can be found on the github README page):

<https://github.com/MASILab/ADSP_AutoQA>

0.) Please try to access this link: **<*URL_FOR_SHARED_GOOGLE_SHEET*>**. Edit access should have been sent via email. Reply if you do not have access or did not receive the email.

1.) Go to the directory containing the tool and run the command:

python3 app_montage.py **</path/to/root/folder>**

2.) Copy and paste the link into a browser (please use google chrome if possible, as it works best on chrome).

3.) **Have a timer prepared to time how long each process takes.** Click on the folder corresponding to your QC ID.

At this point, you should see a selection of 8 different folders, each corresponding to an MRI processing pipeline (1 for SLANT-TICV, 1 for PreQual, 5 for Tractseg, 1 for LungHarmonization). As you have already performed QC on 7 of the 8 pipelines, please ONLY perform QC of the remaining pipeline, **LungHarmonization**. Below are the ONLY instructions you will receive for how to perform the QC; use your own discretion when making decisions based on these rules and these rules alone:

LungHarmonization: You will be assessing whether or not harmonization of Lung CT scans from one type of kernel to another have introduced "anatomical hallucinations" in the CT scans, where "anatomical hallucinations" for this specific QC task are referring ONLY to instances where lung tissue is expanding past the ribcage. Below is an example of one such instance:


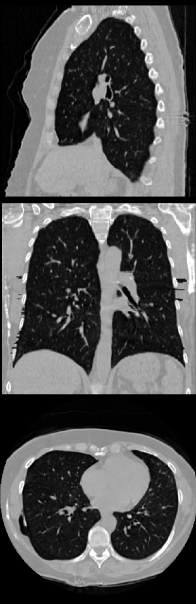


where lung tissue is noticeably hallucinated to be leaking out past the ribcage. While there may be some other artifacts present in the images, such as the "zipper-like" artifact in the back of the person for the image above, these other artifacts are NOT to be counted as hallucinations FOR THIS PARTICULAR QC TASK. Instances where any hallucinations of lung tissue extending past the ribcage are present are "no" results. A "yes" result is a scan absent of this hallucination. Below is an example of a "yes" result where there are no artifacts present of the lung tissue extending past the ribcage:


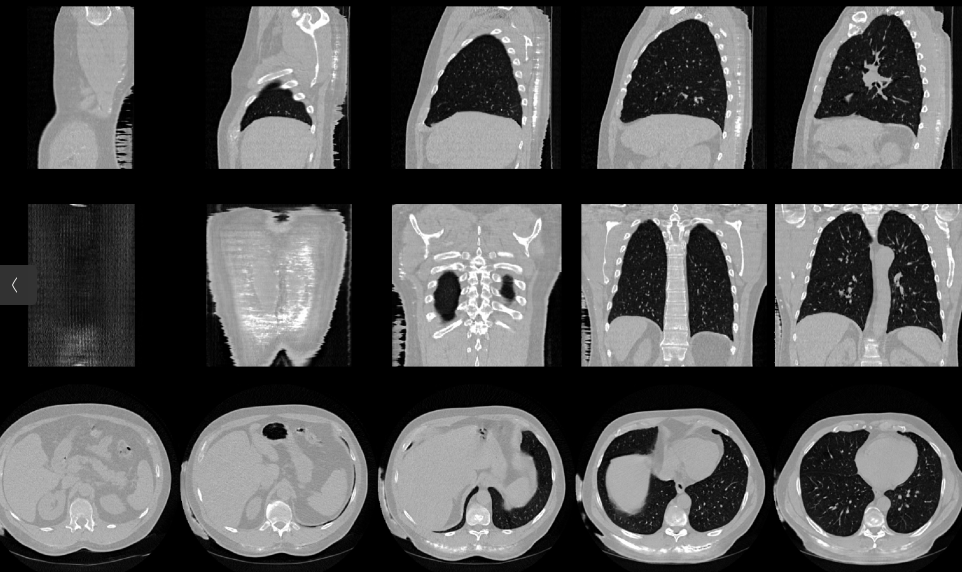


Notice how there are no hallucinations where lung tissue extends past the ribcage, however, there are additional hallucinations that we are not considering for this experiment. Thus, to reiterate, this example is a "yes" result.

If you feel that none of the descriptions above match your interpretation for the quality of the output, you may mark the particular output as "maybe". Please feel free to use the provided text box to give any accompanying text for your decision if you feel it is necessary. Once you are finished with QC of a pipeline, please make sure to record the time that it took to finish the QC of the pipeline here in this google sheet: **<*URL_FOR_SHARED_GOOGLE_SHEET>***

4.) Once you have finished the QC task, please reply to this thread with the message: "Complete!"

**********

## Supplementary Information Section B: Timing Information

**Table ST.B1.** **Pipeline QC Experiment Timing.** Recorded times for QC of each of the respective pipelines, reported as *minutes:seconds*. Note that in addition to different sample sizes for each experiment, some pipeline outputs, such as PreQual, have more information contained in each QC PNG compared to other pipelines.

|  | **PreQual (N=315)** | **SLANT-TICV (N=1000)** | **TractSeg AF right (N=1000)** | **TractSeg CC4 (N=1000)** | **TractSeg SLF left (N=1000)** | **TractSeg TPOSTC right (N=1000)** | **TractSeg CST left (N=1000)** | **Lung CT Harmonization (N=480)** |
| --- | --- | --- | --- | --- | --- | --- | --- | --- |
| **Rater 1** | *7:23* | *3:55* | *10:31* | *9:13* | *5:19* | *20:10* | *7:53* | *40:11* |
| **Rater 2** | *8:37* | *5:32* | *6:05* | *9:31* | *4:23* | *17:10* | *8:01* | *68:08* |
| **Rater 3** | *25:25* | *14:21* | *8:06* | *13:00* | *6:15* | *17:20* | *7:24* | *19:08* |
| **Rater 4** | *13:12* | *4:45* | *12:41* | *11:31* | *5:46* | *20:15* | *9:27* | *39:04* |
| **Average** | ***13:39*** | ***7:08*** | ***9:20*** | ***10:48*** | ***5:25*** | ***18:43*** | ***8:11*** | ***41:37*** |

## Supplementary Information Section C: Additional Figures and Tables

**Table SC.T1.** **Inter-rater variability data inclusion.** Sample sizes for previously-labeled data that were included in the inter-rater variability experiments. “Rejected Data” means that the data were previously labeled as bad quality, whereas “Non-rejected Data” means that the data were considered of sufficient quality. Note that these previous ratings are not to be thought of as ground truth labels, nor are they used that way in the experiments. Rather, they are used solely for the purpose of ensuring that some amount of contentious or questionable data are included for the raters.

|  | **PreQual (N=315)** | **SLANT-TICV**  **(N=1000)** | **TractSeg AF right**  **(N=1000)** | **TractSeg CC4**  **(N=1000)** | **TractSeg SLF left**  **(N=1000)** | **TractSeg TPOSTC right**  **(N=1000)** | **TractSeg CST left**  **(N=1000)** |
| --- | --- | --- | --- | --- | --- | --- | --- |
| **Rejected Data** | *35* | *34* | *64* | *205* | *19* | *602* | *134* |
| **Non-rejected Data** | *280* | *966* | *936* | *795* | *981* | *398* | *866* |


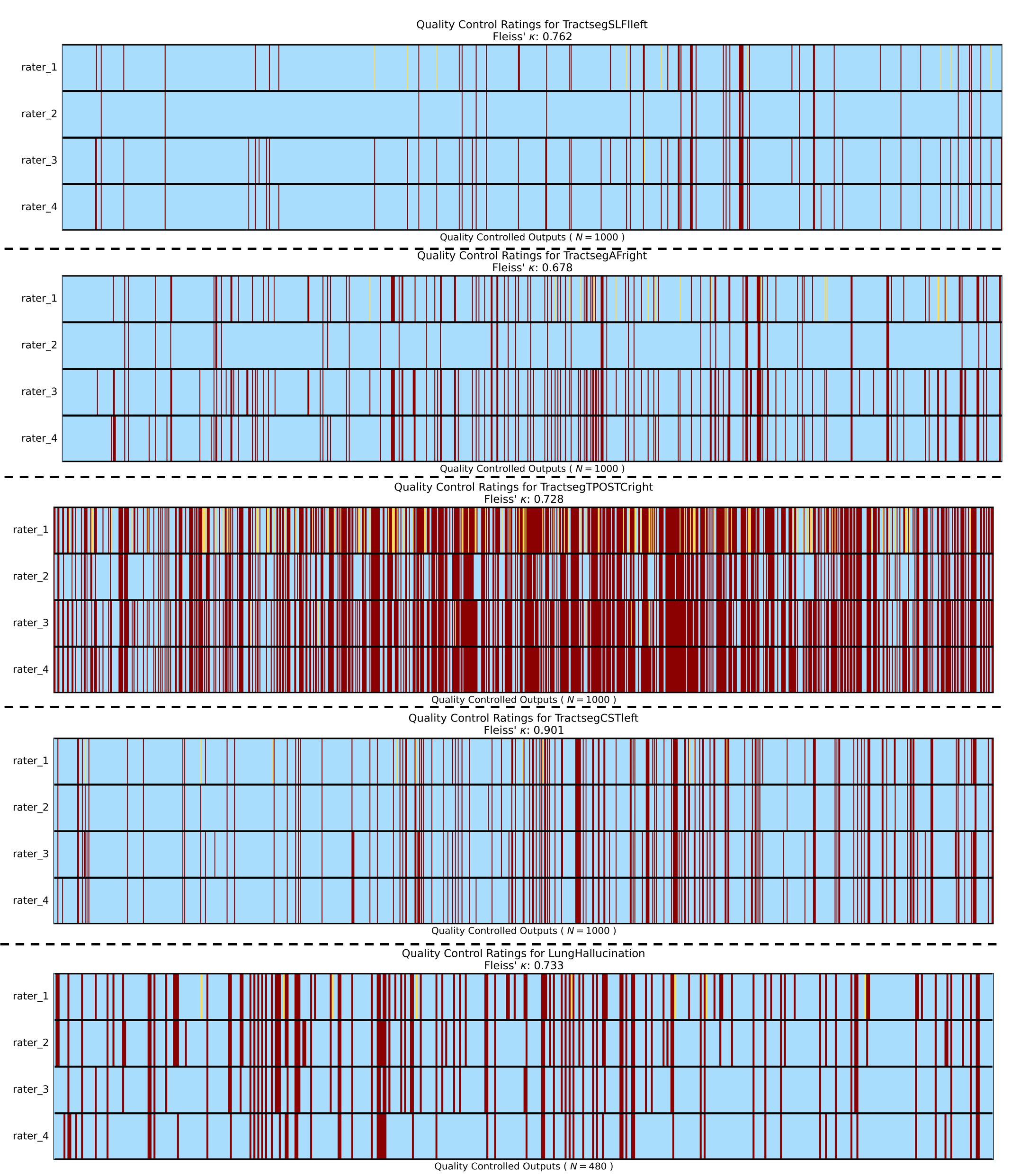


**Figure SC.F1.** **TractSeg inter-rater variability results for four tracts.** Inter-rater variability of the four raters for QC for four of the TractSeg tracts and the Lung CT Harmonization: (top to bottom) SLF left, AF right, TPOSTC right, CST left, and Lung CT Harmonization. The Fleiss’ Kappa scores show high agreement (0.6 – 0.8) between the raters for the SLF left, AF right, TPOSTC right, and Lung CT Harmonization, and very high agreement for the CST left (> 0.8). Blue indicates that the rater selected “yes” for the output while yellow and red indicate selection of “maybe” and “no” respectively.


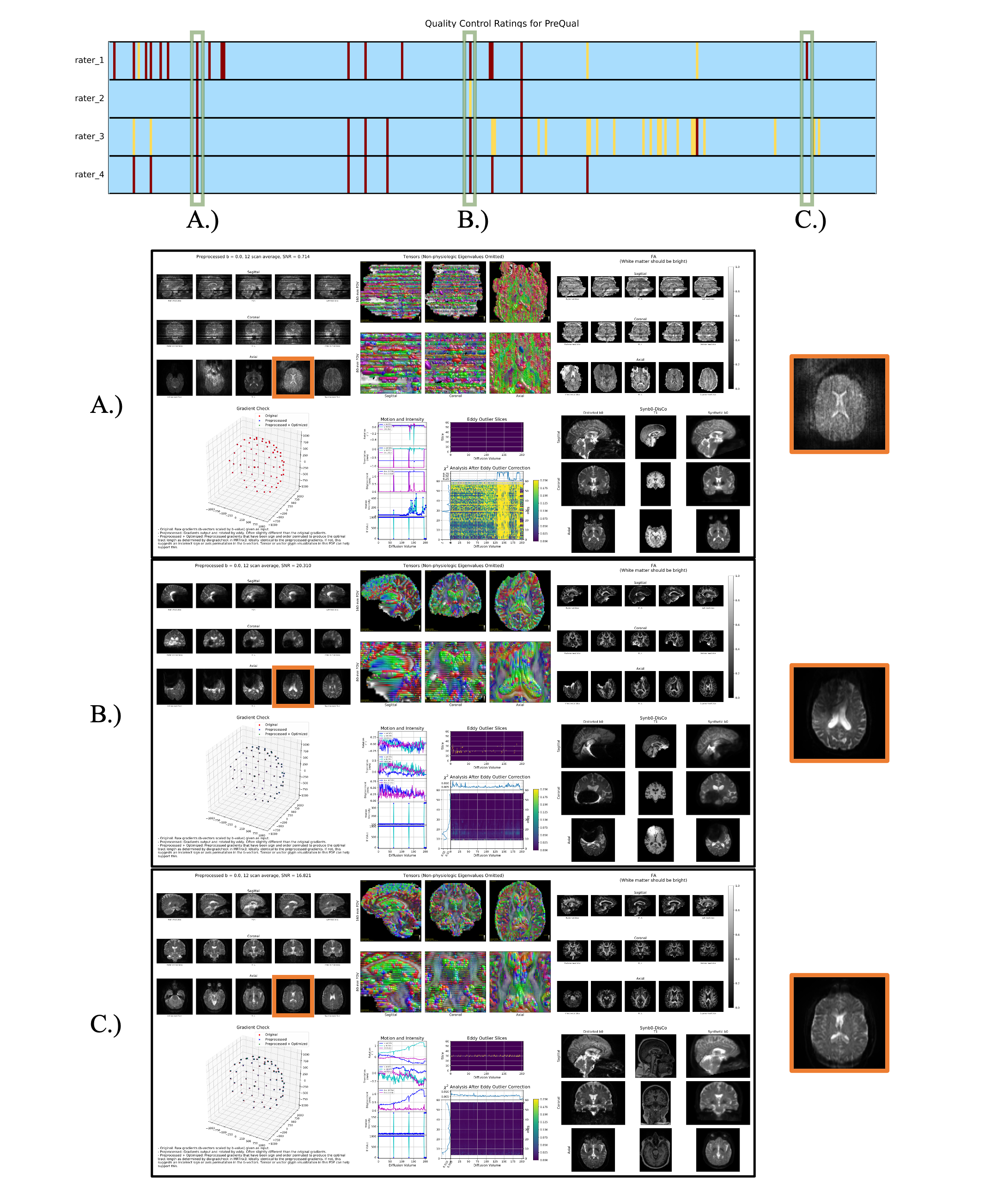


**Figure SC.F2.** **Qualitative results of PreQual inter-rater variability.** For QC of PreQual outputs, decision differences among raters depended on the subtlety of the preprocessing failure or poor data quality. A.) For an output rejected by all raters, the median intensity graph is sporadic, the Chi-squared analysis for scan reconstruction from tensors shows poor similarity, the fitted tensors are sporadic, the FA map is incredibly noisy, and the preprocessed scan has clear artifacts. B.) For an instance when most raters reject an output, there is a clear image artifact present both before and after preprocessing. C.) In an instance where only one rater rejected an output, most steps look acceptable, but close inspection of the preprocessed image and the Synb0-DisCo result indicate that the synthesized b0 image is anatomically inaccurate, causing TOPUP to incorrectly estimate susceptibility-induced distortions.


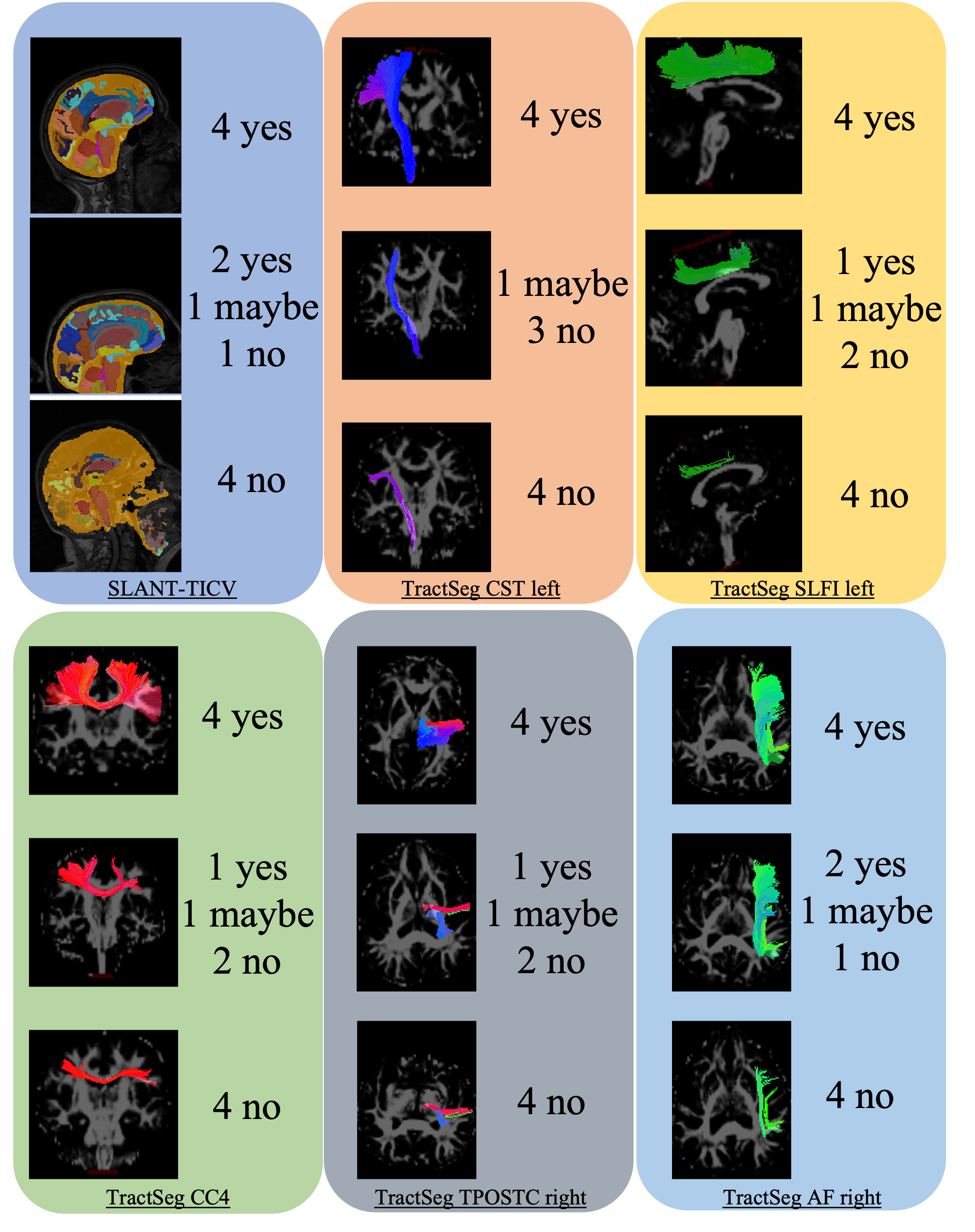


**Figure SC.F3.** **Qualitative results of SLANT-TICV and TractSeg inter-rater variability.** For QC of SLANT-TICV (top left) and TractSeg pipelines (from left to right and top to bottom starting in the top middle: CST left, SLFI left, CC4, TPOSTC right, AF right), outputs with complete agreement across all raters were either clear successes or clear failures. Good SLANT-TICV segmentations showed proper brain parcellation with accurate intracranial vault (ICV) estimation, whereas failures clearly had segmentations outside of the brain. Disputed ratings for SLANT-TICV occurred for instances where the field of view cuts off a small part of the brain or the ICV is overestimated. TractSeg tracts that passed for all raters had a ”full” appearance, whereas failures appeared wispy or were missing some portions of the tract. Disputed ratings for TractSeg occurred when tracts spanned most of the expected region, but not quite the entirety of it.


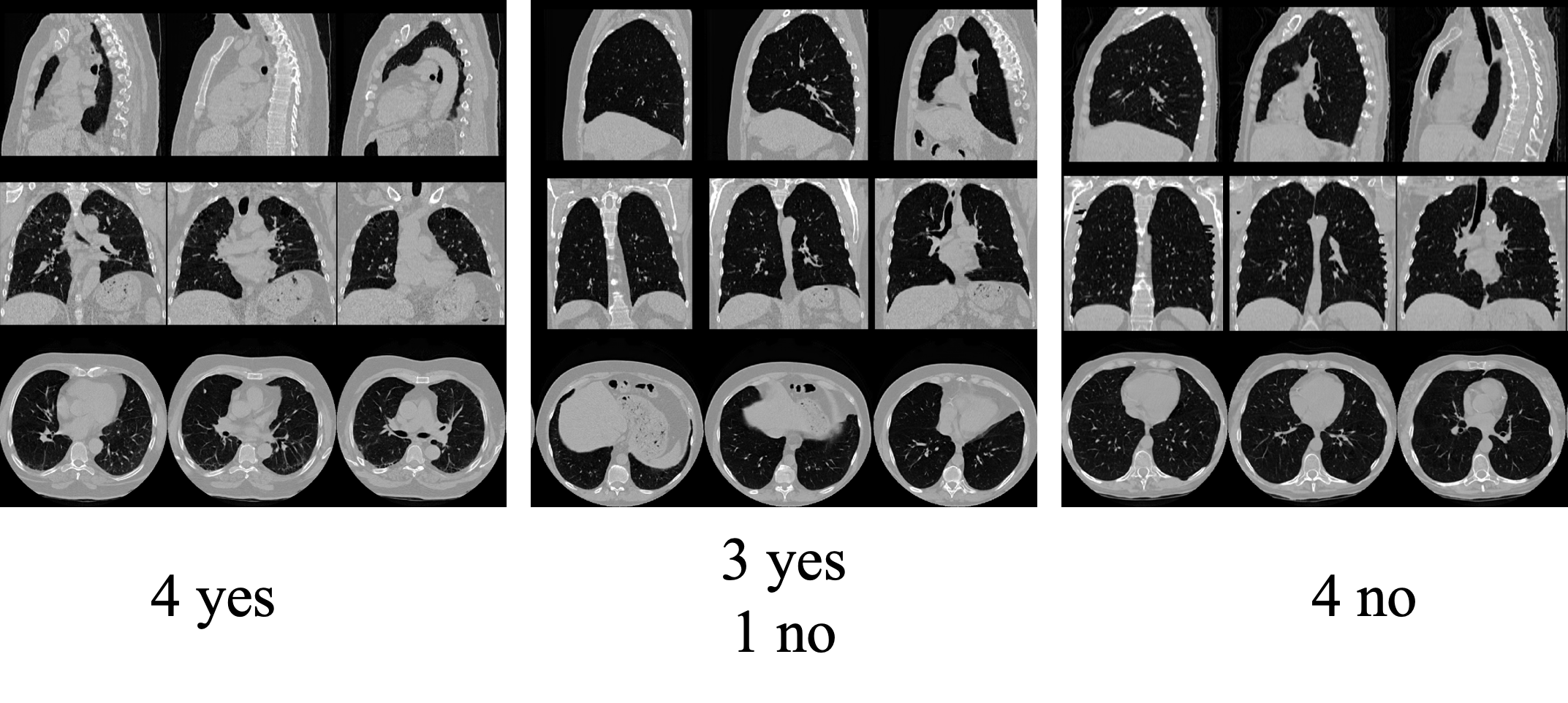


**Figure SC.F4. Qualitative results of Lung CT Harmonization inter-rater variability.** For QC of Lung CT Harmonization outputs, decision differences among raters depended on the subtlety of the hallucinations for lung tissue extending beyond the ribcage. (Left) For an instance where all raters assessed the scan as good quality, the lung tissue is contained within the ribcage. (Center) In a case where only one rater marked the scan as a failed output, lung tissue is very close to the ribcage, which could potentially be marked as a hallucination. (Right) For a scan rejected by all four raters, there are clear hallucinations of lung tissue expanding past the ribcage.


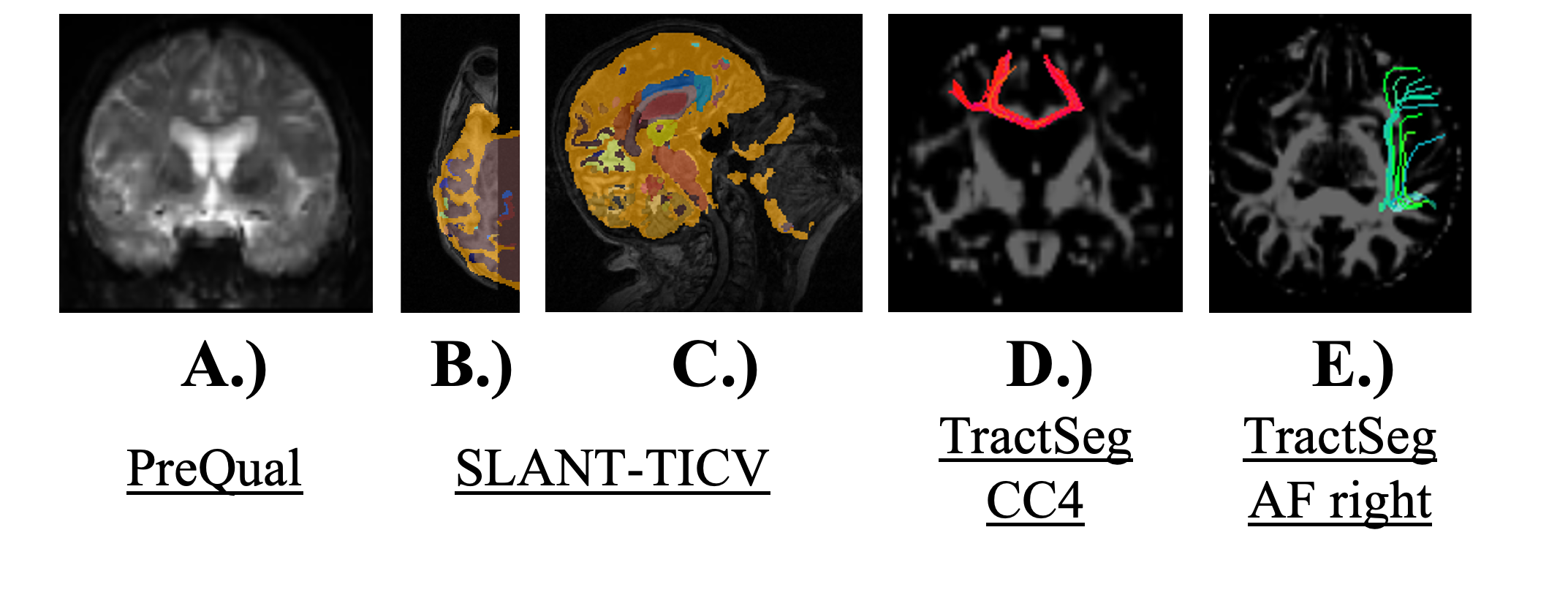


**Figure SC.F5. Examples of bad quality data included in the QC experiments.** A.) One example of a PreQual output where the preprocessing corrections introduced ghosting artifacts in the image. Example SLANT-TICV outputs where B.) the T1w image is only half a brain and C.) the segmentation is well outside the brain. For TractSeg, examples of D.) the CC4 and E.) AF right tracts that are wispy and poor quality.
